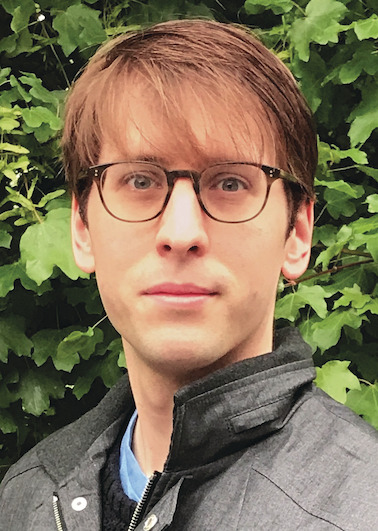# Carbon‐Carbon Bond Formation from Carbon Monoxide and Hydride: The Role of Metal Formyl Intermediates**

**DOI:** 10.1002/anie.202219203

**Published:** 2023-03-02

**Authors:** Joseph M. Parr, Mark R. Crimmin

**Affiliations:** ^1^ Department of Chemistry Molecular Sciences Research Hub Imperial College London 82 Wood Lane Shepherds Bush, London W12 0BZ UK

**Keywords:** Carbon Monoxide, Ethenediolate, Fischer Tropsch, Homologation, Metal Formyl

## Abstract

Current examples of carbon chain production from metal formyl intermediates with homogeneous metal complexes are described in this Minireview. Mechanistic aspects of these reactions as well as the challenges and opportunities in using this understanding to develop new reactions of CO and H_2_ are also discussed.

## Graphical abstract

In this Minireview, we summarise the current examples of carbon chain production from metal formyl intermediates with homogeneous metal complexes. We discuss the mechanistic aspects of these reactions and the challenges and opportunities in using this understanding to develop new reactions of CO and H_2_.

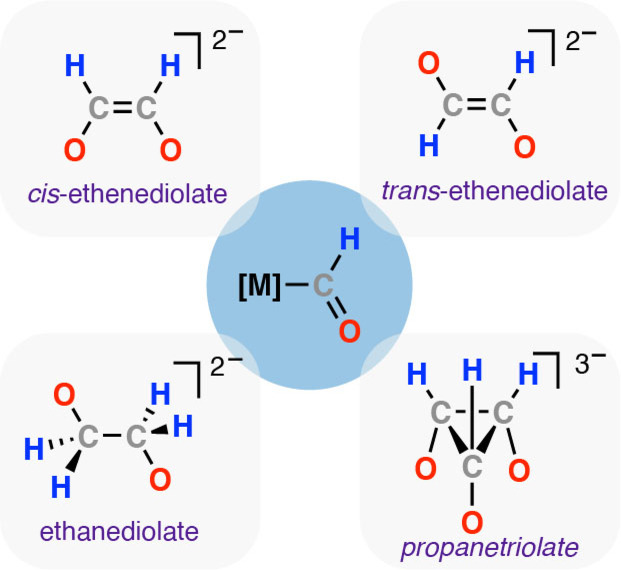

## 1 Introduction

Carbon oxides, such as carbon monoxide (CO) and carbon dioxide (CO_2_), offer an intriguing potential to build carbon‐carbon bonds directly with high atom efficiency. The ideology is inspired by nature, where enzymes are known to use CO_2_ and H_2_O in the production of complex sugars during photosynthesis. Some have suggested that even under prebiotic conditions CO may be one of nature's building blocks for sugars.[^1^, ^2^] These “C_1_ building blocks” are commonly discussed in terms of their toxicity or negative impact as greenhouse gases; their industrial value can be underappreciated.

CO is an abundant, cheap source of carbon and oxygen atoms. It can be readily obtained from fossil fuel sources including coal and natural gas. Many renewable sources of CO gas are also available: biomass, wood waste, and recycled plastics.[^3^, ^4^, ^5^, ^6^, ^7^] Further, CO can be produced from CO_2_ directly by the water‐gas shift reaction [Eq. 1].^[8]^ Key industrial processes use CO as a building block. For example, the Cativa process produces acetic acid by the iridium catalysed carbonylation of methanol at the 500 000‐ton scale annually.[^9^, ^10^] Similarly, the hydroformylation “oxo” process produces aldehydes from reaction of alkenes, CO, and H_2_.^[11]^ The Fischer–Tropsch (FT) process provides liquid hydrocarbons from syngas, mixtures of CO and H_2_, and can be considered as the controlled hydrogenation and homologation of CO. The FT process is typically operated when it is economically viable or necessary due to restricted access to raw materials.^[12]^ For example, the Pearl Gas‐to‐Liquid Factory converts 150,00− barrels per day of natural gas to liquid hydrocarbons and lubricants.^[13]^

(1)

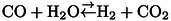



(2)

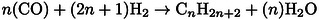



(3)

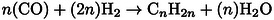




The FT reaction consists of an initiation step, a chain‐growth step, and a termination step. The FT process operates under forcing conditions, commonly 473–623 K, 20–45 bar pressure.[^13^, ^14^] A heterogeneous catalyst comprising a transition metal (M=Co, Fe),^[15]^ a chemical promoter (K_2_O), and a chemically inert structural motif (e.g., SiO_2_, Al_2_O_3_, or MgO) is employed.[^16^, ^17^, ^18^] Incorporation of main group metals into the heterogeneous transition metal catalyst are known to improve activity and selectivity.^[19]^ The primary reaction products are short to medium chain alkanes [Eq. (2)] and alkenes [Eq. (3)] formed alongside other hydrocarbon oxygenates. A Schultz‐Flory distribution of hydrocarbons is obtained (C_10_–C_20_)^[20]^ resulting in the requirement for expensive separation processes and ultimately limiting the proliferation of this process.

The fundamental reactions governing carbon‐carbon bond formation remain a challenge to elucidate, despite the FT process being known for over a century. In all cases, the reaction must proceed via C≡O and H−H bond cleavage, subsequent C−C homologation, and eventual dehydration. Surface bound transition metal carbonyls have been proposed as reaction intermediates during the heterogeneous FT process.^[17]^ Hydrogen activation at the metal surface is proposed to give reactive metal hydrides as the other key intermediate in FT reactions.^[18]^ The combination of these two surface bound moieties underpins chain growth at the catalytic site, potentially via transient metal‐formyl intermediates.

Substantial effort to model the mechanistic processes of FT chemistry have been made using homogeneous metal complexes. These are far more amenable to study than heterogeneous systems, as the nature of the active site and reaction intermediates is readily elucidated by solution and solid‐state characterisation methods.^[21]^ While homogeneous systems are yet to prove highly effective as catalysts for FT,[^8^, ^21^] their study allows elucidation of the fundamental steps for C−H and C−C bond formation from CO and H_2_ (or hydride sites). In this mini‐review, we summarise the current examples of carbon chain production from metal formyl intermediates with homogeneous metal complexes. To date, the formation of both unsaturated and saturated C_2_ motifs along with cyclic C_3_ products has been reported. We discuss the mechanistic aspects of these reactions and the challenges and opportunities in using this understanding to underpin future FT chemistry.

## 2 State‐of‐the‐art

### 2.1 Structural Motifs from CO and H^−^

**Figure 1:**
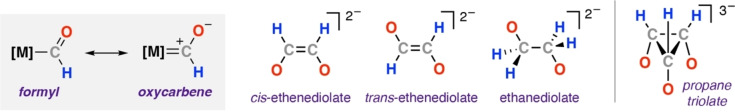
Canonical forms of a metal‐formyl complex and reported structural motifs formed via metal‐formyl intermediates.

The simplest way to combine CO and H^−^ on a metal surface results in the formation of a formyl ligand (CHO^−^). Two related mechanisms can be proposed for formyl generation: an intermolecular insertion of CO into a polarised metal‐hydride bond, or an intramolecular migratory insertion of CO into a metal‐hydride bond at the same metal centre. These mechanisms are closely related, differing only by the formation of a stable metal carbonyl intermediate. Metal‐formyl complexes have been proposed as intermediates in FT processes for both homo‐ and heterogeneous systems.[^8^, ^22^] Despite their importance, the detailed study of transition metal formyl complexes has been hampered by their low stability.^[23]^ Early studies established that these species undergo facile α‐elimination to form the corresponding metal‐hydrido carbonyl complex. This reaction is potentially reversible, though in most cases the hydrido‐carbonyl is thermodynamically favoured.[^24^, ^25^, ^26^, ^27^] The first structurally characterised formyl complex, [ReCp(CHO)(NO)(PPh_3_)], was reported by Gladysz and co‐workers in 1979.^[28]^ We^[23]^ and others^[29]^ have shown that the formyl ligand can be stabilised through resonance with the corresponding oxycarbene structure (Figure 1) or through coordination of the formyl oxygen atom to the metal centre.[^22^, ^30^]

**Figure 2:**
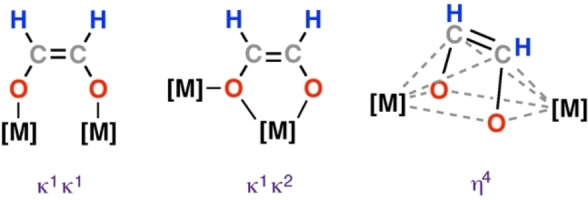
Coordination modes for metal‐coordinated ethenediolate ligands.

Despite their limited stability, metal formyl complexes have been invoked in the formation of several C_2_ and C_3_ products (Figure 1) through further reactions with CO and H_2_ (or hydride sites). The most prevalent products are *cis*‐ethenediolate complexes. These contain a {C_2_H_2_O_2_}^2−^ ligand whose coordination mode is dependent upon the nature of the metal centre and supporting ligand. κ^1^, κ^2^, and η^4^ motifs are known (Figure 2). The related *trans*‐ethenediolate complex is markedly less common. Higher C_3+_ homologues are rare, with a single example of a C_3_ propanetriolate {C_3_H_3_O_3_}^3−^ ligand reported to date. The known structural motifs have been observed across a series of homogeneous models including transition metal, main group, lanthanide, and actinide complexes. Examples of deoxygenated products from these reactions are also limited; however, ethylene, allyloxide, ethylidene, squarate and hexolate production has been reported from reactions of metal‐hydride complexes with CO, with formyl intermediates very likely in all cases.

### 2.2 Ethenediolate Formation

**Scheme 1:**
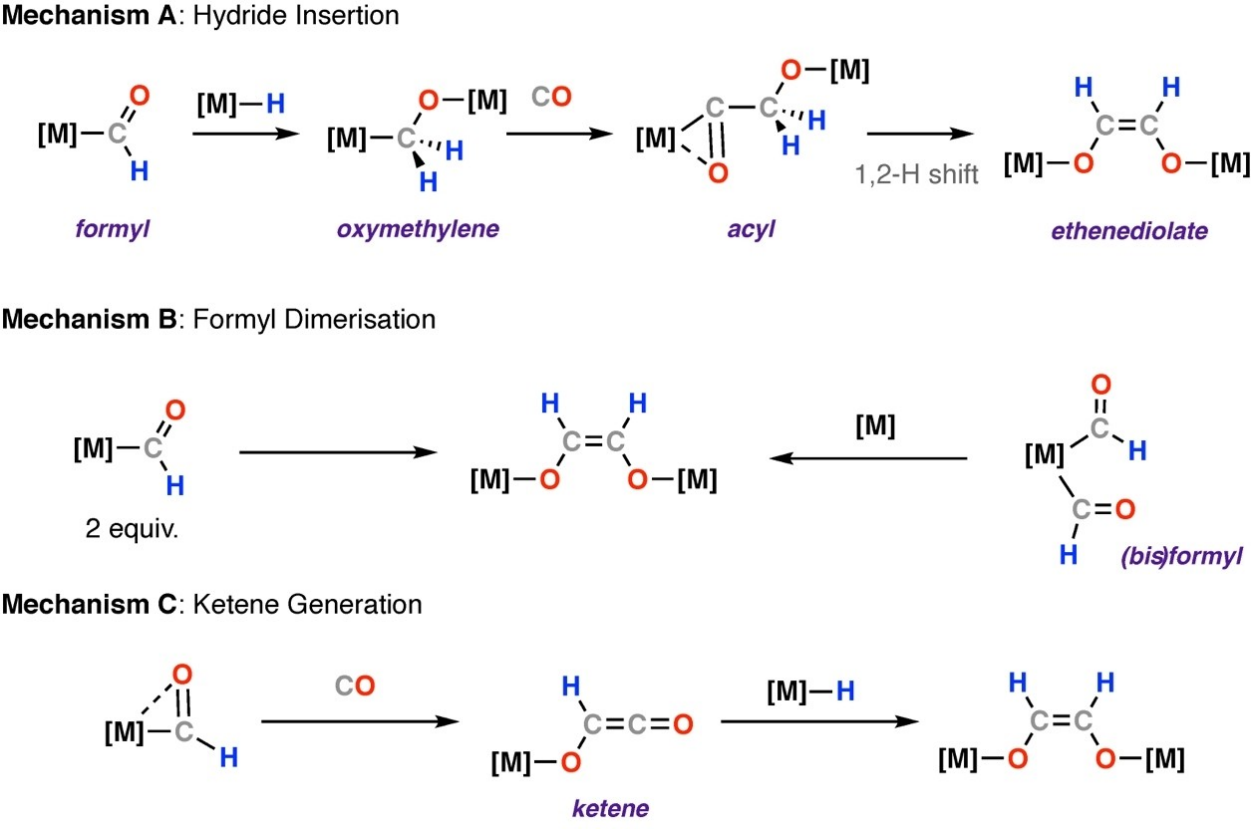
Mechanisms for ethenediolate formation from metal formyl intermediates.

Several reaction mechanisms can be envisioned for ethenediolate formation from 2 CO+H_2_ through metal formyl intermediates. Scheme 1 shows the three most commonly discussed routes for carbon chain growth following generation of a metal formyl, these are: **Mechanism A)** A multistep hydride insertion mechanism involving: (1) hydrometallation of the formyl intermediate to give bridging oxymethylene intermediate; (2) migratory insertion of a second CO molecule into the M−C bond to give an acyl intermediate; (3) 1,2‐hydride shift and rearrangement to give ethenediolate complex;^[31]^ **Mechanism B)** direct dimerisation of formyl ligands, either on two distinct formyl‐complexes or a metal bis‐formyl;^[32]^ **Mechanism C)** CO insertion into the metal‐formyl ligand, generating a C_2_‐ketene intermediate, followed by insertion of the ketene into a second equivalent of M−H to yield the ethenediolate ligand.^[33]^

**Scheme 2:**
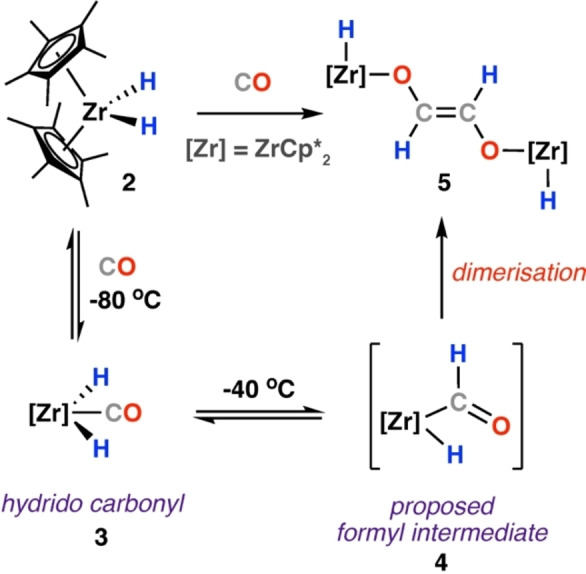
Reductive homologation and hydrogenation of CO by a zirconocene hydride complex **2**.

The first suggestion of metal‐formyl complexes as intermediates in CO homologation relevant to the FT process was reported almost simultaneously by Casey and Bercaw in 1976.[^34^, ^35^] Casey and co‐workers reported a metal‐formyl complex formed by the reaction of a polarised metal‐hydrogen bond with a metal carbonyl complex. Reaction of a series of Fe, Cr and W carbonyl complexes with trialkoxyborohydrides [HB(OR)_3_]^−^ furnished the corresponding metal formyl complexes, identified by characteristic low‐field resonances in the ^1^H and ^13^C NMR spectra. Though no direct C−C bond formation was observed, the authors speculated on the role of formyl complexes as potential intermediates in the first step of catalytic CO homologation with H_2_.^[35]^ At the same time, Bercaw and co‐workers were investigating dinitrogen zirconium complexes, isolating [Zr(Cp*)_2_N_2_]_2_ (**1**). The N_2_ ligand on **1** was readily displaced with CO or H_2_, acting as an entry point into species of relevance to FT catalysis. Reaction of the zirconium hydride [Zr(Cp*)_2_(H)_2_] (**2**) with CO at −80 °C reversibly formed [Zr(Cp*)_2_(H)_2_(CO)] (**3**). NMR analysis indicated a symmetric structure of **3**, with the CO ligand occupying the central equatorial position mutually *cis* to both hydride ligands. Warming solutions of **3** above −50 °C resulted in formation of the *trans*‐ethenediolate complex [{Zr(Cp*)_2_H}_2_(μ‐OCH=CHO)] (**5**). **5** was characterised by diagnostic NMR resonances, δ_H_=5.73 (s, 1H) and 6.55 (s, 1H) ppm, and IR absorption bands (ν_Zr‐H_=1580; ν_C‐O_=1205 cm^−1^). The authors suggested a mechanism for ethenediolate formation that proceeded via a transient formyl intermediate (**4**). **4** was proposed to form by insertion of CO into one of the *cis* Zr−H bonds and undergo a subsequent non‐reversible reaction to form the ethenediolate **5** (Scheme 2).^[34]^ The putative intermediate **4** was not observed spectroscopically or characterised.

**Scheme 3:**
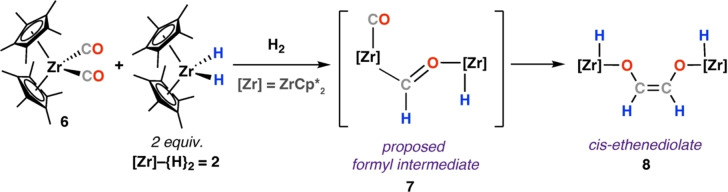
Reaction of zirconocene carbonyl (**6**) and hydride (**2**) complexes to provide cis‐ethenediolate complex **8**.

Subsequent work detailed the reduction of zirconium‐coordinated CO ligands with **2** (Scheme 3).^[31]^ Reaction of [Zr(Cp*)_2_(CO)_2_] (**6**) with **2** under an atmosphere of H_2_ yielded the *cis*‐isomer of ethenediolate complex **8**. It is noteworthy that the analogous reaction in the absence of H_2_ gave a mixture of products. The reaction was predicted to proceed via a chelating formyl ligand bridging the two zirconium centres (**7**). A series of metal carbonyls (M=W, Cr, Mo, Nb) were shown to react analogously with **2**, providing isolable bridging formyl complexes but no C−C coupled products.^[36]^

**Scheme 4:**
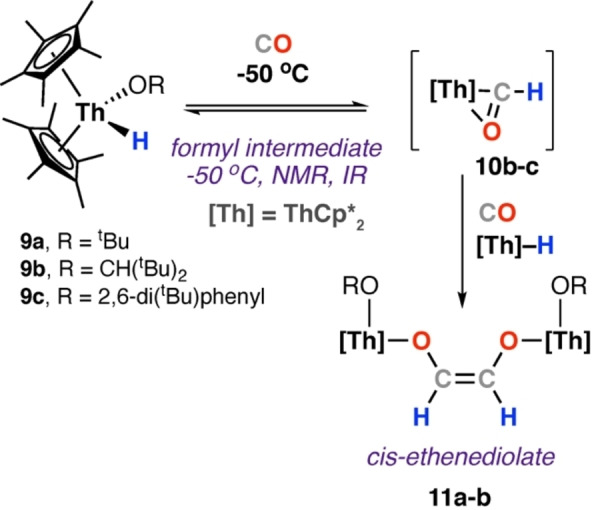
Reversible insertion of CO into the Th−H bond of pentamethyl cyclopentadienyl thorium complexes **9 a**–**c** and onward reaction of formyl intermediate **10 b**–**c** to yield cis‐ethenediolates **11 a**–**b**.

In 1981, Marks and co‐workers reported the first spectroscopic characterisation of a metal formyl complex as an intermediate in ethenediolate formation (Scheme 4).[^37^, ^38^] Reaction of thorium hydride complex [Th(Cp*)_2_(OR)(H)] (**9 a**–**b**) with CO at 25 °C yielded the *cis*‐ethenediolate complex **11 a**–**b**. The *cis*‐geometry of the ethenediolate ligand was identified by NMR spectroscopy techniques and ^13^C labelling studies. The rate of reaction was impacted by the relative steric bulk of the alkoxide ligand. Reaction of **9 b**–**c** with CO at −78 °C gave transient formyl complexes **10 b**–**c**. **10 b**–**c** were characteristic at −50 °C by diagnostic ^1^H and ^13^C NMR resonances (δ_H_=15.2 and 14.7 ppm; δ_C_=372 and 360 ppm, for **10 b** and **10 c** respectively) and IR stretching frequencies (ν_CHO_=1477 cm^−1^ for **10 b**). At higher temperatures intermediate, **10 b** went on to form an ethenediolate complex, while **10 c** did not. The overall reaction of **9 b** to form ethenediolate **11 b**, via observable formyl complex **10 b**, was the first direct evidence for metal formyl complexes as intermediates in CO homologation. Reaction of the less bulky parent actinide complex [Th(Cp*)_2_(μ‐H)H]_2_ with CO at −78 °C yielded the *cis*‐ethenediolate complex [{Th(H)(Cp*)_2_}_2_(μ‐C_2_H_2_O_2_)] **12**; in this case no intermediate was observable spectroscopically.[^38^, ^39^]

#### 2.2.1 Mechanism A: Hydride Insertion

The most invoked mechanism for ethenediolate formation from formyl intermediates is through the hydride insertion **Mechanism A**. This mechanism was proposed by Bercaw in their original work but with limited experimental or computational support for the steps following generation of the formyl intermediate.[^31^, ^40^]

**Scheme 5:**
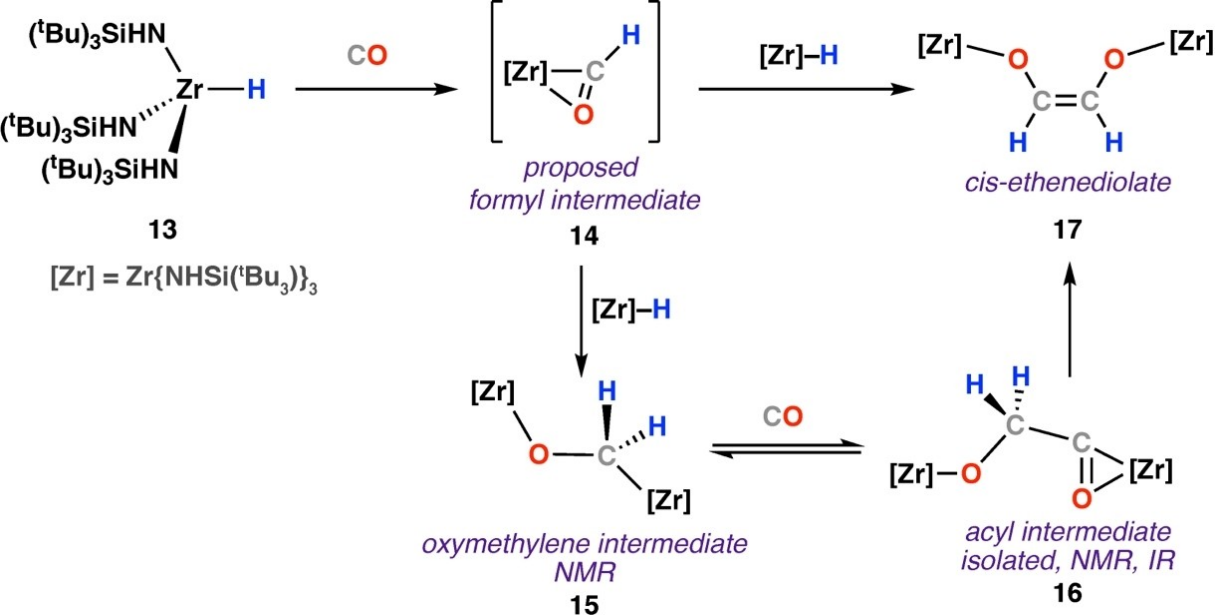
Reaction of terminal zirconium hydride complex **13** with CO. Oxymethylene (**15**) and acyl (**16**) intermediates were identified during formation of cis‐ethenediolate **17**.

In 1991, Wolczanski and co‐workers reported the reaction of CO with zirconium hydride complex [Zr(NHSi ^t^Bu_3_)_3_H] (**13**) to form an ethenediolate species (Scheme 5).^[41]^ While in this case the formyl intermediate **14** could not be observed, its formation is underpinned by the discoveries with closely related Zr and Th metallocene systems described above.[^31^, ^37^] Nevertheless, data to support the formation of two downstream intermediates in the hydride insertion mechanism was collected. Specifically, the oxymethylene complex **15** was characterised by NMR spectroscopy δ_H_=4.38 ppm, δ_C_=84.8 ppm; **15** had a lifetime of c.a. 45 minutes in C_6_D_12_ solution at 12 °C. Onward carbonylation of **15** at 25 °C yielded the spectroscopically observed acyl intermediate [{Zr(NHSi^t^Bu_3_)_3_}_2_(μ‐OCH_2_CO)] (**16**), and, ultimately, formation of *cis*‐ethenediolate complex [{Zr(NHSi^t^Bu_3_)_3_}_2_(μ‐C_2_H_2_O_2_)] (**17**). Stoichiometric reaction of **13** with 0.5 equiv of CO in n‐hexane yielded the acyl intermediate **16** as an isolable colourless solid in 79 % yield. Ethenediolate **17** was characterised by diagnostic vinylic NMR spectra resonances at δ_H_=5.62 and δ_C_=127.94 ppm.

**Scheme 6:**
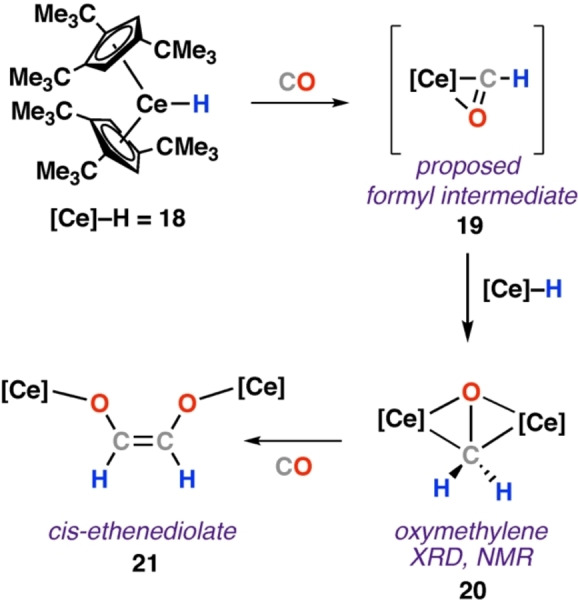
Reaction of terminal cerium‐hydride complex **18** with CO to give oxymethylene intermediate **20** and ultimately cis‐ethenediolate complex **21**.

Andersen and co‐workers reported the reaction of monomeric [Ce(Cp′)_2_H] (**18**; Cp′=1,2,4‐trimethylcyclopentadienyl) with CO yielded the corresponding *cis*‐ethenediolate complex **21** (Scheme 6).^[42]^ Conducting the reaction in n‐pentane solution led to precipitation of the bridging oxymethylene intermediate [{Ce(Cp′_2_)}_2_‐(μ‐CH_2_O)] (**20**), isolated as orange crystals. **20** was predicted to form from transient formyl intermediate **19**. The bridging dimeric structure of **20** was confirmed by X‐ray diffraction analysis. Onward reaction of **20** with CO gave ethenediolate **21**, identifying it as an intermediate in CO homologation. Density Functional Theory (DFT) calculations, conducted by Eisenstein, were used to gain insight into the system. Although this study focused on onward reaction of **19** with H_2_ to give the cerium methoxide complex **22**, the initial steps of the calculated mechanism are of the most relevance to this discussion. **18** was simplified to the cyclopentadienyl analogue [Ce(Cp)_2_H] (**23**) for computational cost. Reaction of **23** with CO was calculated to proceed via CO coordination to cerium and subsequent migratory insertion of CO into the Ce−H bond. A modest energy barrier transition state (Δ*G* ^≠^_298K_=6.1 kcal mol^−1^) was located for an overall exergonic process to yield the μ^2^‐formyl complex [Ce(Cp)_2_(η^2^‐CHO)] (**24**; Δ*G* ^o^_298K_=−13.9 kcal mol^−1^). Onward reaction with an additional equivalent of **23** to give oxymethylene complex **24** was discussed, but further energy profile not calculated in this instance.

**Scheme 7:**
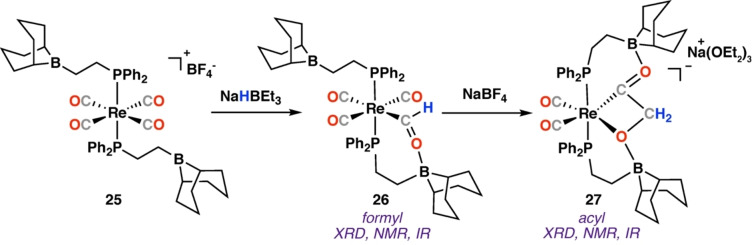
Reaction of rhenium carbonyl complex **25** with a nucleophilic hydride source (NaHBEt_3_).

In 2008, Labinger, Bercaw, and co‐workers reported the reductive coupling of CO mediated by a hydride source and a rhenium carbonyl complex with pendant Lewis acids (Scheme 7). Reaction of a rhenium phosphinoborane complex (**25**) with CO in the presence of one equivalent of NaHBEt_3_ provided rhenium‐formyl complex **26** in quantitative yield; **26** was characterised by a downfield singlet in the ^1^H NMR spectra (δ_H_=13.95 ppm) and single crystal X‐ray diffraction analysis. Addition of a second equivalent of NaHBEt_3_ provided the rhenium cyclic acyl complex **27**, wherein the acyl unit is stabilised by coordination of the two Lewis‐acidic boryl groups. The structure of **27** was confirmed by NMR spectroscopy and single crystal X‐ray diffraction. Rearrangement to the predicted ethenediolate was not observed in this instance (likely due to the stabilising Lewis acid sites), though the fundamental steps of C−C formation follow those outlined in **Mechanism A**. The predicted oxymethylene intermediate was discussed by the authors, but not observed in this instance.

**Scheme 8:**
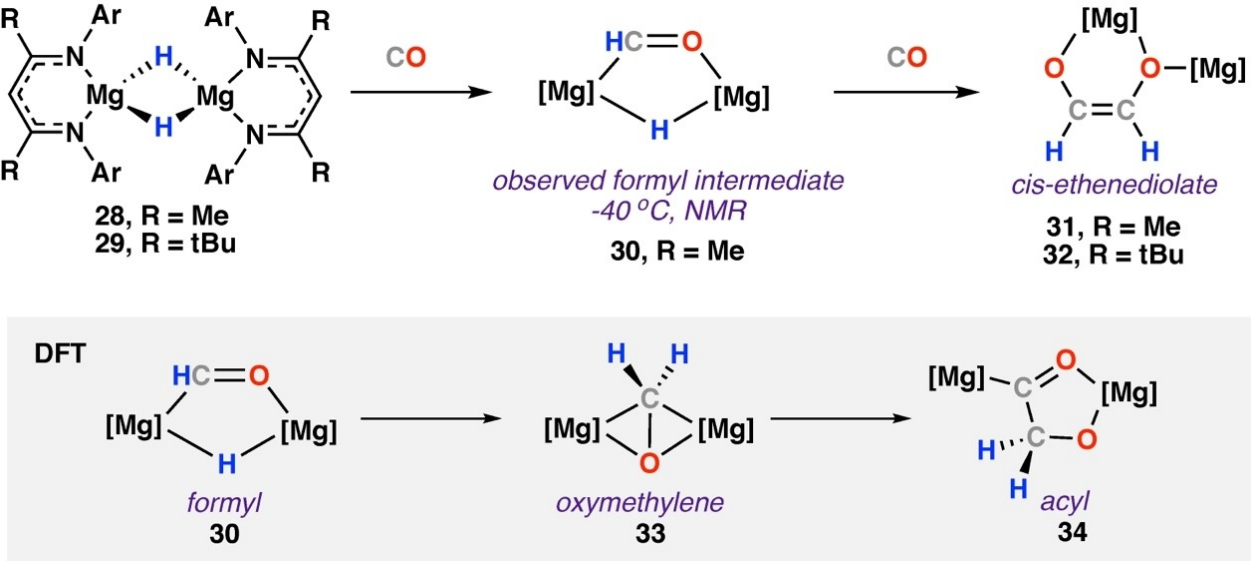
Reaction of a dimeric bridging magnesium hydride complex **28**–**29** with CO. DFT calculated pathway (grey) identified oxymethylene (**33**) and acyl (**34**) intermediates during ethenediolate formation **31**–**32**. Ar=2,6‐diisopropylphenyl.

In 2015 Hill and co‐workers^[43]^ and Jones, Stasch, Maron and co‐workers^[44]^ simultaneously reported the reaction of a β‐diketiminate stabilised magnesium hydride reagent with CO (Scheme 8). Reaction of [Mg{(μ‐H){CH{C(CH_3_)NDipp}_2_}]_2_ (Dipp=2,6‐diisopropylphenyl) (**28**) with CO yielded the *cis*‐ethenediolate complex [{Mg(CH{C(CH_3_)NDipp}_2_)}_2_(μ‐C_2_H_2_O_2_)] (**31**). **31** adopts an unusual asymmetric bridging mode, wherein one of the Mg centres is bound through chelation to both ethenediolate oxygen atoms. The longer Mg−O bonded oxygen is further bound to the other trigonal Mg centre. **31** was characterised by single crystal X‐ray diffraction. Running the reaction at −40 °C provided spectroscopic evidence for the anticipated formyl intermediate. The structure of **30** was proposed based on NMR spectroscopic data: δ_H_=14.08; δ_C_=358.9 ppm.

DFT calculations support the generation of a formyl complex as an intermediate in CO homologation and follow the mechanism presented in **Mechanism A**. Insertion of CO into the Mg−H bond of **28** suggested the formation of a transient formyl intermediate via a barrierless reaction. An intramolecular hydromagnesiation proceeds via a modest (Δ*H* ^≠^=14.8 kcal mol^−1^) energy barrier to give the bridged oxymethylene complex **33** (Δ*H* ^≠^=−15.8 kcal mol^−1^). A second CO insertion and subsequent 1,2‐H shift yielded the identified ethenediolate complex through an overall exergonic reaction (Δ*H*°=−82.9 kcal mol^−1^ from free **28** and CO).

**Scheme 9:**
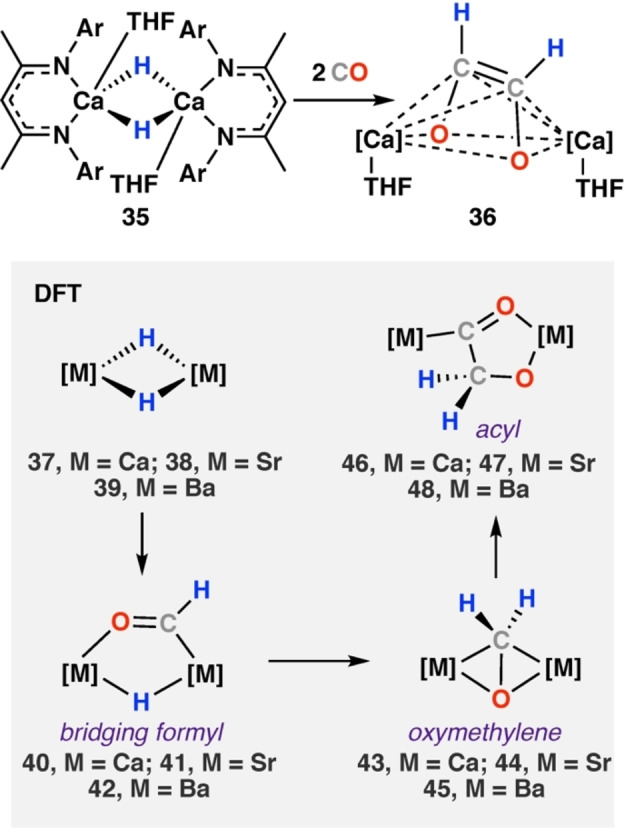
Reaction of β‐diketiminate stabilised calcium hydride dimer **35** with CO. DFT calculated pathway (grey) supports formyl, oxymethylene, and acyl intermediates for Group 2 metals Ca, Sr, and Ba. Ar=2,6‐diisopropylphenyl.^[45]^

Exchanging the methyl groups on the β‐diketiminate backbone to t‐butyl was anticipated to change the rate of reaction with CO and possibly allow identification other reaction intermediates. Reaction of [Mg(μ‐H){CH{C(^t^Bu)NDipp}_2_}]_2_ (**29**) with CO at −90 °C yielded ethenediolate (**32**) as the sole product. Hill and co‐workers also extended this work to the heavier group 2 metals.^[45]^ Reaction of the β‐diketiminate calcium hydride [Ca(μ‐H)(THF){CH{C(CH_3_)NDipp}_2_}]_2_ (**35**) with CO immediately provided ethenediolate complex **36** (Scheme 9). **36** was characterised by diagnostic vinylic resonances in the NMR spectra (δ_H_=5.00 ppm, δ_C_=134.9 ppm). An X‐ray diffraction analysis of **36** showed a new bonding motif for the ethenediolate ligand. **36** is a dimeric calcium species in which two seven‐coordinate calcium centres are bridged by a dianionic *cis*‐ethenediolate ligand. The *cis*‐ethenediolate ligand bonds to each calcium centre via symmetric bridging η^4^‐interactions. The change in hapticity is proposed to occur because of the larger ionic radius of Ca^2+^ compared to Mg^2+^ (0.99 and 0.65 Å, respectively).

DFT calculations suggest a role for a formyl intermediate in the formation of the calcium ethenediolate.^[45]^ The THF‐free monomeric calcium hydride complex [CaH{CH{C(CH_3_)NDipp}_2_] (**37**) was used for the calculation. The yet hypothetical diisopropylphenyl β‐diketiminate stabilised strontium (**38**) and barium (**39**) hydride dimers were also calculated for comparison.^[46]^ In all cases, coordination of CO to the hydride dimer and subsequent migratory insertion of CO into the M−H bond provided transient η^2^‐C,O‐formyl complexes (**40**–**42**). **40**–**42** undergoes further insertion reactions to give bridging oxymethylene (**43**–**45**) and then acyl (**46**–**48**) intermediates. A 1,2‐hydride shift reaction provides the *cis*‐ethenediolate complex. Energetic rearrangement to the observed 7‐coordinate calcium complex **36** was not calculated. The overall process was exergonic for all metals and deduced to be marginally more exothermic down the group (Δ*H*°=−82.9, −97.0, −99.8, −101.1 kcal mol^−1^, for Mg, Ca, Sr, Ba, respectively).

We recently reported reaction of magnesium hydride dimer **28** with a series of metal carbonyl complexes (M=Cr, Mn, Fe, Co, Rh, W, Ir).^[23]^ In all cases, transition metal formyl complexes were obtained with the formyl ligand trapped as part of a chelating structure. Eight complexes were crystallographically characterised, giving the first series of well‐defined metal‐formyl complexes. Solution NMR spectroscopy identified the formyl unit (δ_H_=13.05–15.11 ppm; δ_C_==240–310 ppm), confirmed by HSQC spectroscopy. In most cases, the C−H formyl stretch could be observed in the IR spectra (2546–2635 cm^−1^).

**Scheme 10:**
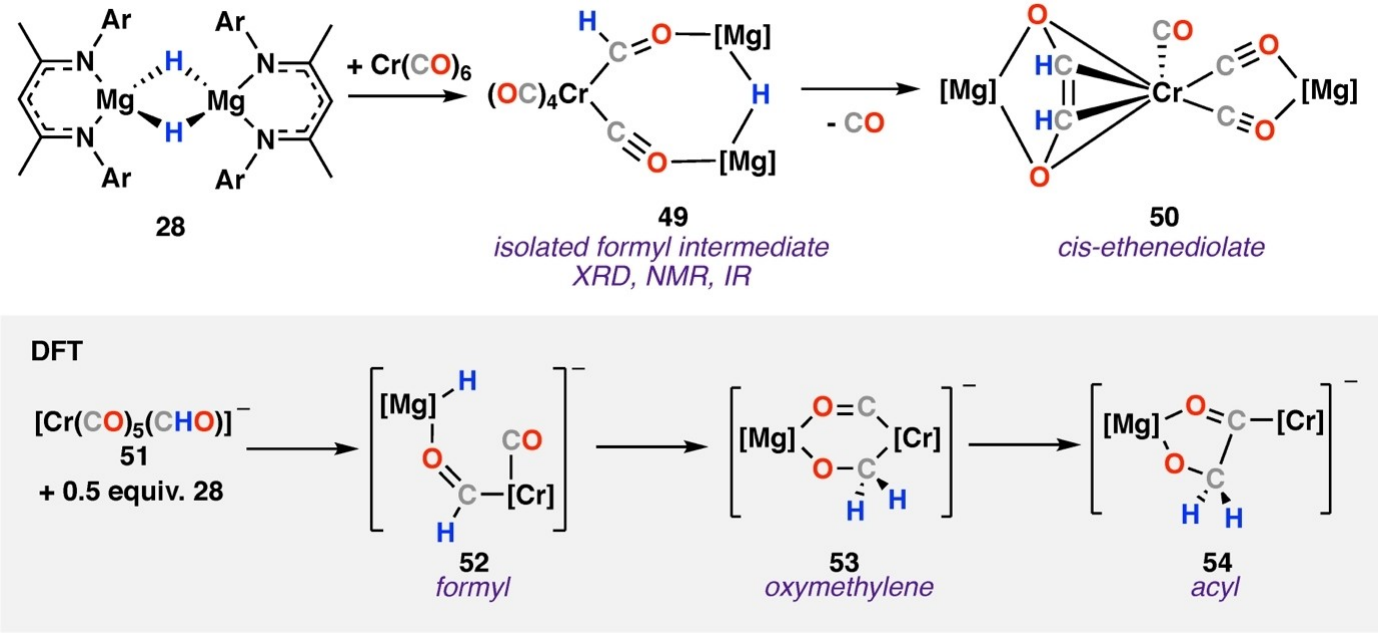
Reaction of magnesium hydride dimer **28** with chromium hexacarbonyl to provide isolable formyl intermediate (**49**) and cis‐ethenediolate **50**. DFT calculated pathway (grey) identified oxymethylene (**53**) and acyl (**54**) intermediates.

Onwards reaction of chromium formyl complex **49** results in C−C bond formation and formation an isolable ethenediolate species **50** (Scheme 10). The ethenediolate ligand in **50** bridges chromium and magnesium centres, binding to chromium through a η^4^‐interaction, reminiscent of the bonding in calcium complex **36**. DFT calculations were performed on a model chromium anion system. The calculated mechanism is consistent with C−C bond formation occurring by stepwise process by **Mechanism A**. Based on the calculation, either step (1) or (3) could be rate determining, proceeding via modest energy transition states: Δ*G* ^≠^_298K_=19.7 and 19.0 kcal mol^−1^ respectively. The modest barrier for transformation of the formyl complex **52** to oxymethylene complex **53** is consistent with the isolable nature of the chromium formyl. C−C bond formation in step (2) occurred via a low transition state (Δ*G* ^≠^_298K_=0.7 kcal mol^−1^) to yield **54**. The overall ethenediolate formation from the chromium formyl anion **51** and magnesium hydride monomer **28** was exergonic (Δ*G*°_298K)_=−37.1 kcal mol^−1^).

#### 2.2.2 Mechanism B: Formyl Dimerisation

**Scheme 11:**
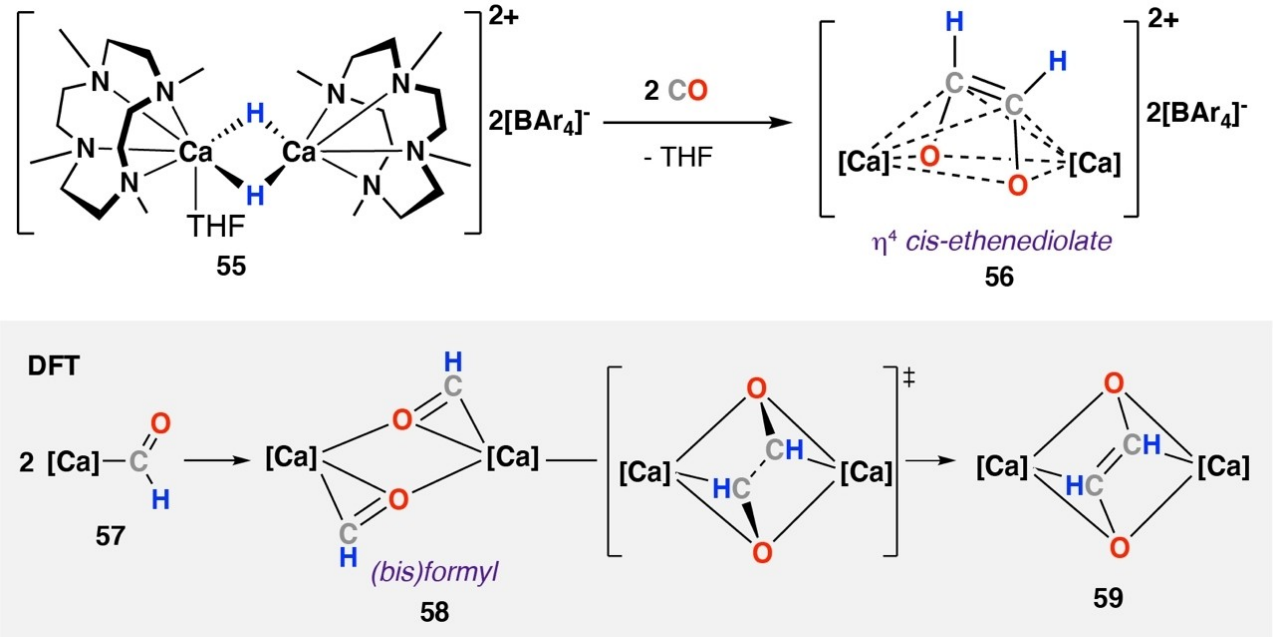
Reaction of N,N,N,N‐macrocycle stabilised calcium hydride complex **55** with CO. DFT calculations (grey) identify bridging formyl intermediate (**58**) during dimerisation to provide calcium‐ethenediolate complex **56**.

In 2020, Okuda and co‐workers reported reaction of a cationic calcium hydride supported by an *N,N,N,N*‐type macrocycle, [{Ca(μ‐H)(Me_4_TACD)}_2_(THF)][BAr_4_]_2_ (**55**) (Me_4_TACD=1,4,7,10‐tetramethyl‐1,4,7,10‐tetraazacyclododecane; BAr_4_=B(C_6_H_3_‐3,5‐Me_2_)_4_) with CO (Scheme 11).^[32]^ After 5 minutes at 25 °C in THF solution, full consumption of **55** was observed spectroscopically and formation of *cis*‐ethenediolate complex [{Ca(Me_4_TACD)}_2_(μ‐C_2_H_2_O_2_)][BAr_4_]_2_ **56**. **56** was fully characterised and X‐ray diffraction analysis showed η^4^‐binding of the bridging ethenediolate ligand to each calcium centre. In contrast to the examples discussed above, DFT calculations support a formyl dimerisation mechanism for formation of ethenediolate **59**, as outlined in **Mechanism B**. Dissociation of dimeric calcium hydride **55** and CO insertion into Ca−H bond is suggested to lead to two equiv. of a mononuclear calcium formyl intermediates **57**. Direct coupling of these intermediates by a formyl dimersation is calculated to occur through a modest energy barrier (Δ*H* ^≠^=+20.2 kcal mol^−1^) to provide **58** and ultimately **59** in an overall exergonic process (Δ*H*°=−100 kcal mol^−1^). Direct imerization of two anionic formyl ligands is likely stabilised in this instance by the highly electropositive calcium centre, potentially facilitating this unusual reaction pathway. A related pathway has been proposed for alkynide dimerisation at a calcium centre.^[47]^

**Scheme 12:**
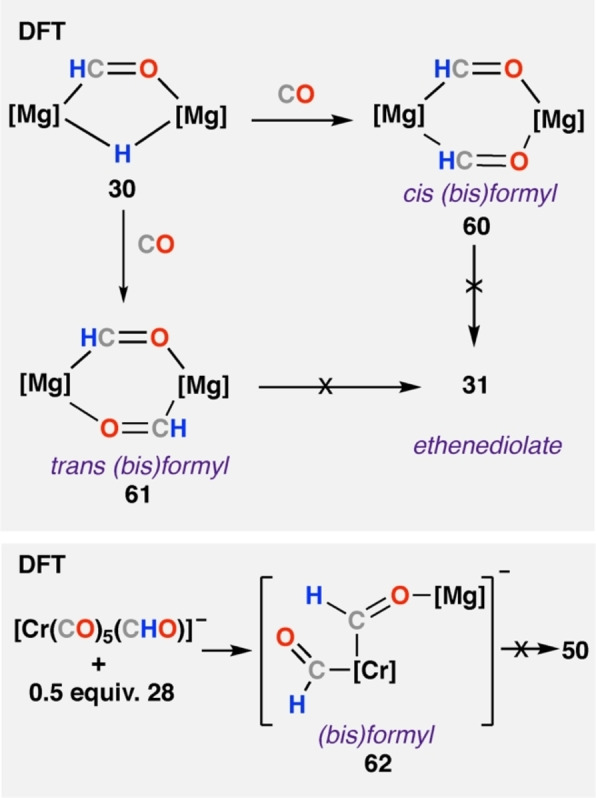
Reported DFT calculated pathways with (*bis*)formyl dimerisation mechanism for ethenediolate formation.

The dimerisation of formyl intermediates has been investigated computationally for other systems. Mechanisms toward magnesium ethenediolate **31** through *bis*(formyl) intermediates were explored computationally (Scheme 12a).^[44]^ Addition of CO to observed bridging formyl hydride complex **30** to give either cis‐(**60**) or trans‐(**61**) *bis*(formyl) intermediates were explored computationally. In both cases, the authors note no route to the observed ethenediolate **31** and higher energy barriers compared to the hydride insertion mechanism discussed in Scheme 9. In our study on ethenediolate formation at Cr—Mg complexes, DFT calculations suggest that direct dimerisation of a putative *bis*(formyl) intermediate (**62**) is a high energy process (Δ*G*°>50 kcal mol^−1^), strongly suggesting that this mechanism is not in operation (Scheme 12b).^[23]^

#### 2.2.3 Mechanism C: Ketene Generation

**Scheme 13:**
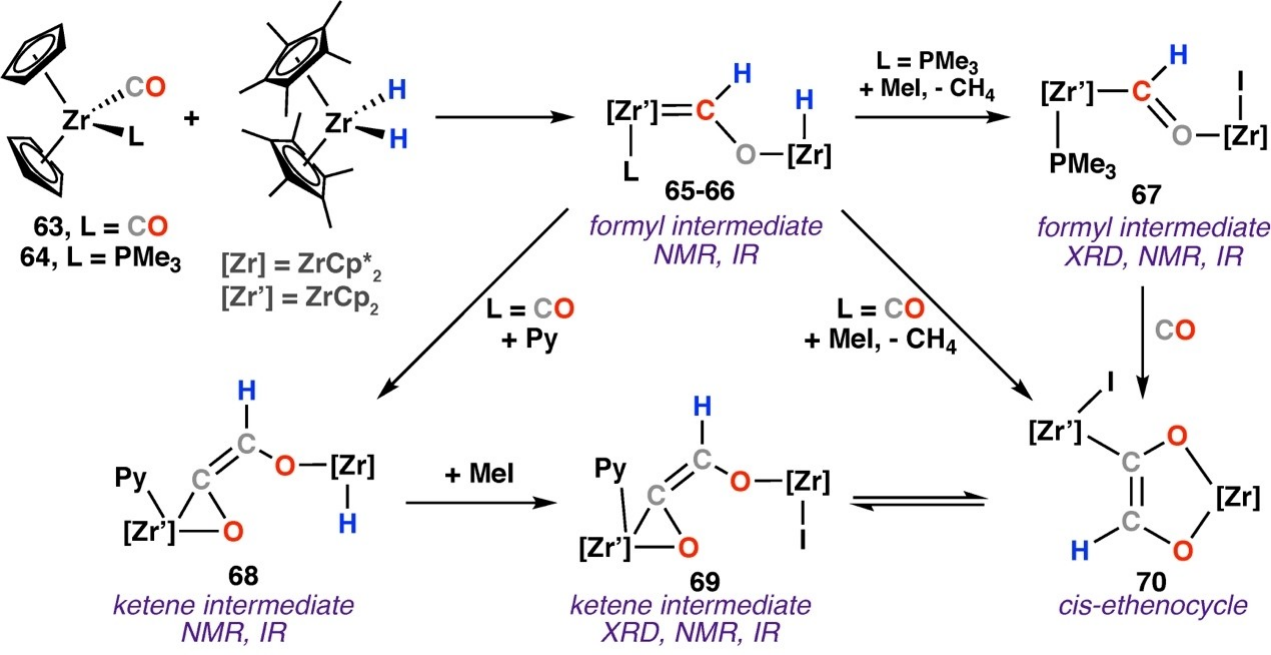
Reaction of zirconium hydride complex **12** with zirconocene carbonyl complexes **63**–**64**. Formyl (**65**–**67**) and ketene (**68**–**69**) intermediates were identified during ethenocycle (**70**) formation.

In 1984, Bercaw and co‐workers reported reaction of **6**, **2**, and H_2_ to give the *cis*‐ethenediolate product **8** (Scheme 3).^[33]^ In efforts to stabilise a proposed formyl intermediate, analogous complexes with less sterically bulky ligands were prepared. Reaction of [Zr(Cp)_2_CO(L)] (L=CO, **63**; L=PMe_3_, **64**) with [Zr(Cp*)_2_(H)_2_] (**2**) at −78 °C yielded the bridging formyl complexes **65**–**66**. **66** is remarkably stable, even in solution at 25 °C. **65** and **66** were characterised by diagnostic NMR data for the formyl moiety ranging δ_H_=11.29–11.58 ppm and δ_C_=287.5–295.0 ppm. Formyl C−H stretches were identified in the IR spectra at 2755 cm^−1^. Reaction of bridging formyl complex **66** with methyl iodide yielded [{Zr(Cp)_2_(PMe_3_)}(μ‐CHO){Zr(Cp*)_2_I}] (**67**) as green crystals, eliminating methane gas detected in the ^1^H NMR spectra. The structure of **67** was confirmed by single crystal X‐ray diffraction analysis. Onward reaction of **67** with CO yields the ethenediolate zirconocycle **70**, characterised by multinuclear NMR, IR, and X‐ray diffraction analysis. Reaction of [{Zr(Cp)_2_(CO)}(μ‐CHO){Zr(Cp*)_2_H}] (**65**) with methyl iodide also yielded ethenediolate **70**. The reaction was predicted to proceed via migratory insertion of CO into the Zr−C bond of bridging formyl complex **65**. Remarkably, addition of pyridine to the **65** gave the CO inserted ketene intermediate **68**. **68** is characterised by diagnostic NMR resonances for the ketene unit (δ_H_=6.18 ppm; δ_C_=165.7 ppm). Addition of methyl iodide to ketene complex **68** resulted in formation of ketene intermediate **69** and **70** (Scheme 13). This seminal work is an exceedingly rare example in which formyl and ketene intermediates are identifiable and characterised during carbon chain formation from CO and hydride sites and the fundamental steps are related to **Mechanism C** for ethenediolate formation.^[48]^

#### 2.2.4 cis‐ to trans‐Isomerisation of an Ethenediolate

**Scheme 14:**
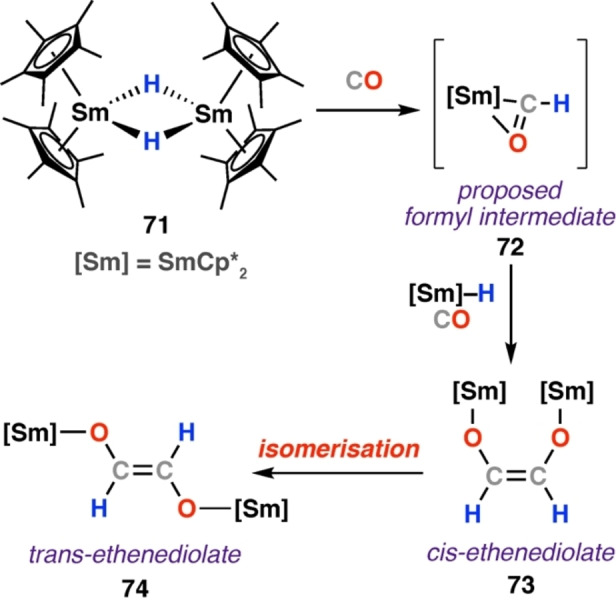
Reaction of samarium hydride dimer **71** with CO. Cis‐ethenediolate product **73** was observed to isomerise to the corresponding trans‐isomer **74**.

The first example of ethenediolate isomerisation was documented by Evans and co‐workers in 1985 (Scheme 14).^[49]^ Reaction of samarium hydride dimer [Sm(Cp*)_2_(μ‐H)]_2_ (**71**) with CO at 25 °C gave the *cis*‐ethenediolate complex **73**, isolated as the triphenylphosphine oxide (TPPO) adduct *cis*‐[{Sm(Cp*)_2_(TPPO)}_2_(μ‐OCH=CHO)] (**75**). **73** isomerised to the *trans*‐isomer (**74**) over a period of several hours to days, with rates depending on solution concentration. A bridging acyl complex was identified as a likely intermediate during isomerisation, with the argument that the *cis* is the kinetic and the *trans* the thermodynamic product discussed. Both *cis*‐ and *trans*‐isomers were fully characterised by multinuclear NMR and IR spectroscopy, and single crystal X‐ray diffraction. Samarium formyl intermediate **72** was proposed as a likely intermediate in ethenediolate formation via a hydride insertion mechanism.

The cerium ethenediolate (**21**) was shown to non‐reversibly convert to the more thermodynamically stable *trans*‐isomer (**76**), over a period of seven months at 100 °C or two weeks at 190 °C under vacuum. Both isomers were fully characterised including by single crystal X‐ray diffraction analysis.^[42]^

### 2.3 Ethanediolate Formation

**Scheme 15:**
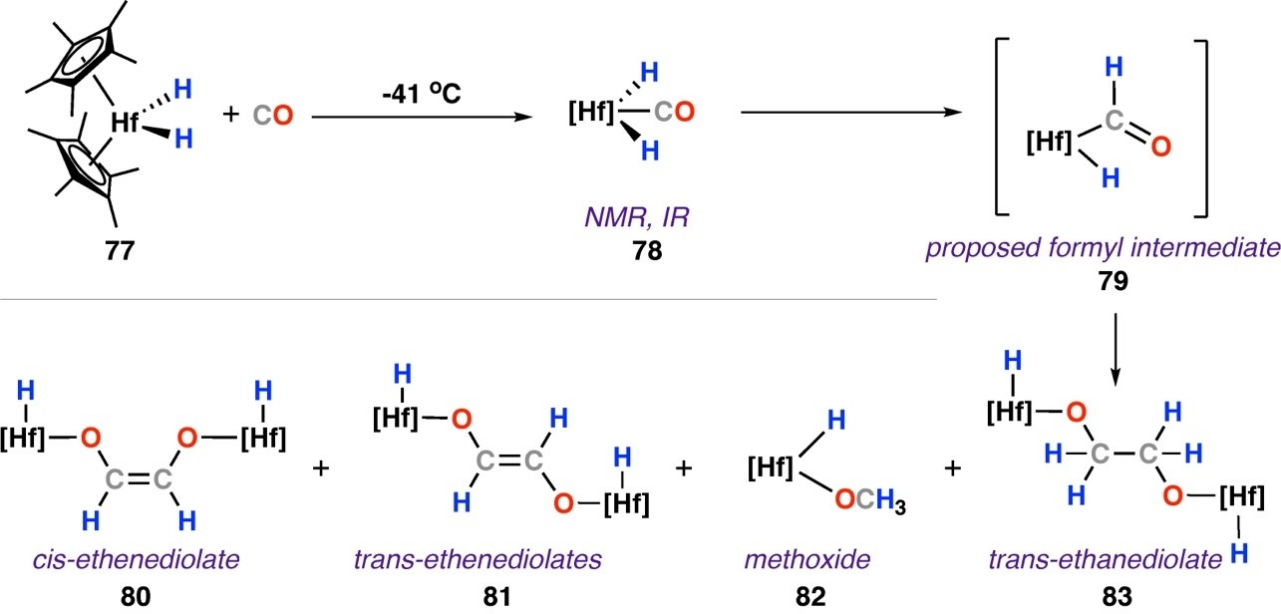
Reaction of hafnium hydride complex with CO to give a mixture of ethenediolate (**80**–**81**), methoxide (**82**) and ethanediolate (**83**) products.

In 1985, Bercaw and co‐workers extended their studies to hafnium complexes. [Hf(Cp*)_2_(H)_2_] (**77**) was prepared through reduction of the parent chloride complex [Hf(Cp*)_2_Cl_2_] with n‐butyl lithium under an atmosphere of dihydrogen.^[50]^ At −41 °C, **77** reacts with CO to form the carbonyl dihydride [Hf(Cp*)_2_(CO)(H)_2_] (**78**); as with **3**, low temperature solution NMR and IR spectroscopy indicate a symmetric coordination of CO to Hf. Solutions of **78** warmed above −10 °C yield a complex mixture of products, including: methoxide, *cis*‐ and trans‐ethenediolate, and ethanediolate hafnium complex (**80**–**83**; Scheme 15). In contrast, reaction of carbonyl complex [Hf(Cp*)_2_(CO)_2_] with metallocene hydrides **2** and **77** formed the expected *cis*‐ethenediolate complexes only. The formation of the ethanediolate species as a minor component of these reactions was unexpected but provides an example of generation of a saturated C_2_ product from CO and hydride sites.

### 2.4 Propanetriolate Formation

**Scheme 16:**
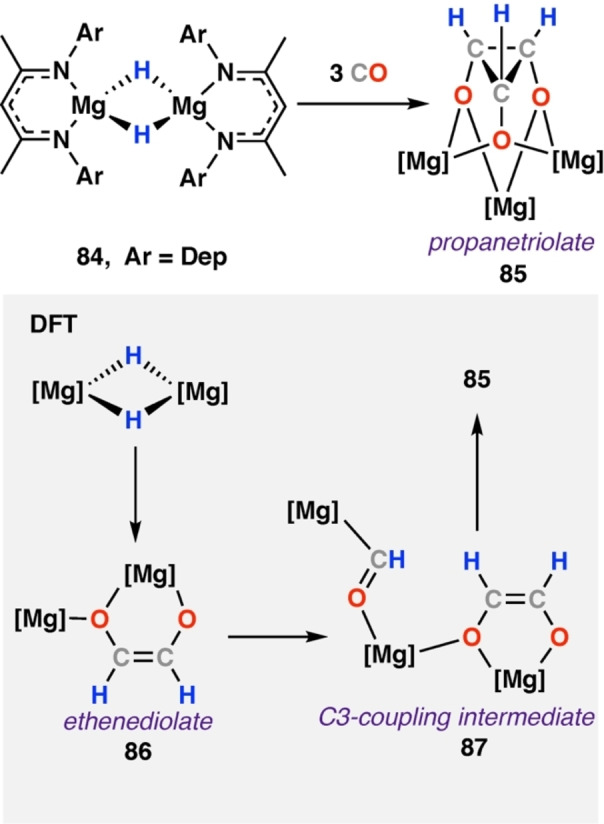
Reaction of a less sterically bulky β‐diketiminate stabilised magnesium hydride complex **84** with CO to give the rare C_3_ propanetriolate complex **85**. Dep=2,6‐diethylphenyl.

Formation of C_3_ or higher products proposed to result from reactions of formyl intermediates with CO and H_2_ (or hydrides) is exceptionally rare. Jones and co‐workers have observed the formation of one such species on reaction of CO with a magnesium hydride complex.^[44]^ The steric influence of the β‐diketiminate ligands were shown to be crucial in determining the selectivity of the reaction. Addition of CO to [Mg(μ‐H){CH{C(CH_3_)NDep}_2_]_2_ (Dep=2,6‐diethylphenyl) (**84**) gave the rare C_3_ homologated propanetriolate product [{Mg{CH{CN(CH_3_)Dep}_2_}_3_(μ‐C_3_H_3_O_3_)] (**85**, Scheme 16). **85** was fully characterised by diagnostic NMR resonances (δ_H_=1.18 ppm; δ_C_=47.7 ppm, for C_3_H_3_O_3_ ligand) and X‐ray diffraction analysis. DFT calculations supporting formation of **85** from the corresponding ethenediolate **89**. Onward reaction of ethenediolate with an additional equivalent of magnesium formyl complex proceeded via **87** to the observed propanetriolate in an overall exergonic process (Δ*H*°=−51.6 kcal mol^−1^ from ethenediolate **86**). DFT calculations for the formation of the related more sterically bulky diisopropyl analogue of **85**, namely [{Mg{CH{C(CH_3_)NDipp}}_2_}_3_(μ‐C_3_H_3_O_3_)] (**88**) were shown to be not kinetically viable, supporting the observed selectivity.

## 3 Summary and Perspective

The 50 years of research described in Section 2 has resulted in a reasonable understanding for the mechanistic steps that combine CO and H_**2**_ (or hydrides) at metal sites to give ethenediolate ligands.[^51^, ^52^, ^53^, ^54^] Metal‐formyl complexes have been identified as transient intermediates and mechanisms for ethenediolate formation proposed. Most data support **Mechanism A** described in Scheme 1 via oxymethylene and acyl intermediates. Despite these findings, examples of well‐defined reactions that form C_3+_ homologues are uncommon. Equally rare are examples of deoxygenation reactions that increase the ratio of C : O to greater than one. These steps, broadly the propagation sequence in FT chemistry, remain of significant interest and arguably the next focus point for the field. It is likely that the current approach of developing homogeneous models, will ultimately shed light on these remaining questions and some selected examples described briefly below support this assumption and provide motivation for further study.

**Longer Carbon Chains**: Reactions of metal‐hydride complexes with CO to give higher (>C_3_) coupled products have been achieved using multimetallic lanthanide and transition metal hydride complexes. C_4_ squarate^[55]^ and C_6_ hexolate^[56]^ ligands have been reported by reductive coupling of CO using titanium‐ and tantalum‐hydride complexes respectively. Though in neither case were formyl or other intermediates identified, the fundamental C−C coupling steps likely proceed via formyl intermediates and subsequent loss of dihydrogen. These successes give precedent for future work in this field and point to multimetallic hydride complexes as attractive candidates for further CO homologation.[^57^, ^58^]

**Figure 3:**
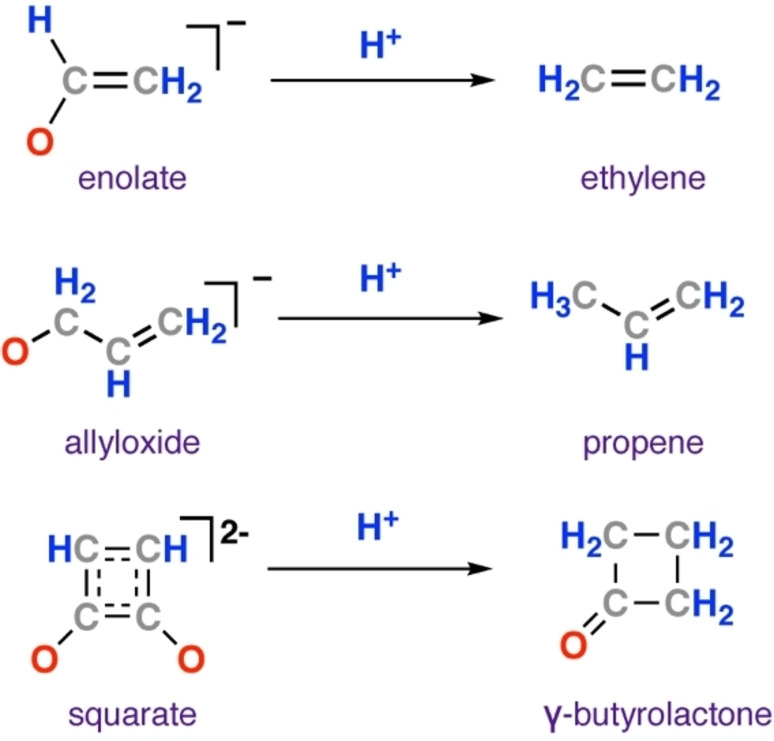
Other products formed from reaction of metal‐hydride complexes with CO.

**Dehydration/Deoxygenation**: A modest number of metal‐hydride complexes have been reported to react with CO to yield deoxygenated ligands, wherein the C : O ratio is greater than one. Ethylidene,^[59]^ enolate,^[60]^ allyloxide,^[61]^ and squarate^[55]^ ligands have been reported from reaction of multimetallic hydrides with CO. In the latter three cases, ethylene,^[60]^ propene,^[61]^ and γ‐butyrolactone^[55]^ were successfully liberated from the metal centre, respectively (Figure 3). In all these instances, cooperative multimetallic complexes were employed (M=Ta, Ln, Y, Ti). Many of the known reactions occur at highly electropositive metals and are likely thermodynamically driven through the formation of strong M−O bonds. However there is extremely limited mechanistic evidence for how these dehydration or deoxygenation events occur.

In the longer term, understanding the fundamental steps for the coupling reactions of CO and H_**2**_ (or hydride sites) at metal centres has the potential to lead to the rational design of homogeneous FT catalysts. Controlling the selectivity of the FT process, and removing the need for energy‐intensive separations, remains challenging. Homogeneous systems have the potential to address this problem while providing access to a diverse range of carbon chain building blocks.

## Conflict of interest

The authors declare no conflict of interest.

## Biographical Information

*Joseph Parr: Graduated from the University of Leeds in 2018 with an MChem(Int), spending a year abroad at the University of South Carolina. He completed a MSc in organometallic chemistry at the University of Southern California in 2020, under the supervision of Prof. Inkpen. He has recently enjoyed a JSPS Short‐Term Fellowship, working with Prof. Nakao at Kyoto University. Joe is currently a third year PhD student at Imperial College London, researching CO homologation and carbon‐carbon bond activation using main‐group hydride reagents*.

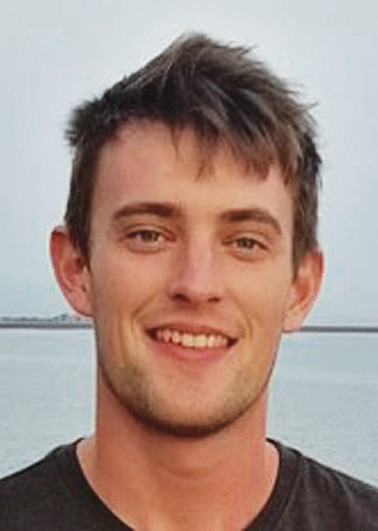

## Biographical Information

*Mark Crimmin: Graduated from Imperial College London in 2004 and completed a MSc by research in organic synthesis at Bristol University under the supervision of Prof. Aggarwal. He received his PhD in main group chemistry and catalysis from Imperial College London in 2008 supervised by Prof. Mike Hill and Prof. Tony Barrett. In the same year, he was awarded a Royal Commission for the Exhibition of 1851 research fellowship which he took to UC Berkeley to study with Prof. Bob Bergman and Prof. Dean Toste. In 2011, he returned to London as a Royal Society University Research Fellow, initially at UCL and now back at Imperial. He was appointed as a lecturer in 2011, Senior Lecturer in 2016, Reader in Organometallic Chemistry in 2019, and full Professor in 2021*.